# Hybridization of CMIP6 and spatiotemporal models for assessing solar energy dynamics and transition risks in Guangxi under “dual-carbon” goals

**DOI:** 10.1016/j.isci.2026.114690

**Published:** 2026-01-14

**Authors:** Yisong Han, Xiangling Tang, Wei Li, Siyi Hu

**Affiliations:** 1 College of Earth Sciences, Guilin University of Technology, Guilin, Guangxi 541000, China

**Keywords:** Environmental science, Energy engineering, Energy Resources

## Abstract

Under the global dual-carbon goals, assessing regional solar potential is vital for the energy transition. This study evaluates solar resource potential and spatiotemporal redistribution risks in Guangxi, China, using ECMWF ERA5 and CMIP6 multi-scenario data. We develop an interactive spatiotemporal regression model by integrating optimal parameter geographic detectors with geographically weighted regression to quantify drivers' synergistic effects. Key findings: (1) solar resources exhibit strong path dependency, with the highest growth rate occurring under the medium-emission scenario due to the atmospheric purification effect.; (2) dominant drivers shift with scenarios: topography (low emissions), cloud-aerosol interactions (medium), and multi-factor synergy (high); and (3) The resource center migrates from the southwestern coast to the northeastern interior, with rising spatial-instability risks. This work supports optimized solar deployment and regional energy transition in Guangxi.

## Highlights

• Developed a framework linking pattern diagnosis, drivers, and probabilistic risk
• Fastest solar growth in medium-emission scenario due to “air-cleansing effect”
• Dominant drivers shift from topography to synergy as carbon scenarios intensify
• A new paradigm for assessing subtropical energy-climate risks in complex terrain

## Graphical abstract

**Figure:**
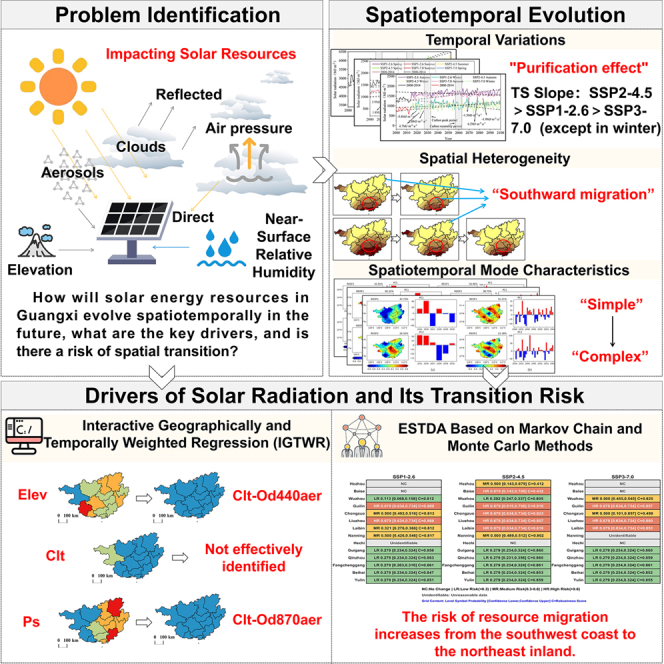

## Introduction

As global climate change mitigation efforts deepen, building a low-carbon energy system has become a core pathway to promoting sustainable development.^1^^,^^2^ Against this backdrop, China’s proposed “Dual-Carbon” goals—achieving carbon peaking before 2030 and carbon neutrality before 2060—align with the global trend and inject strong momentum into global climate governance.^3^^,^^4^ Per the latest reports from authoritative bodies such as the International Energy Agency (IEA), China has become a key driver of the global energy transition: its 2024 clean energy investment exceeded $625 billion, doubling since 2015 and accounting for roughly one-third of the global total.^5^^,^^6^ Such substantial input has effectively fueled the leapfrog development of clean energy, particularly wind and solar power.^7^^,^^8^ By the end of 2024, the nation’s cumulative installed wind and solar capacity hit 1.41 billion kilowatts, accounting for ∼42% of its total installed capacity, meeting the 2030 planning target six years early.^9^^,^^10^ Of this, cumulative photovoltaic (PV) capacity reached 88.6 GW, representing 47% of China’s total clean energy installed capacity. This not only far surpasses concurrent wind power capacity (52.1 GW) but also solidifies PV as the largest single energy source in China’s clean energy system, with its core status growing increasingly prominent.^11^

However, behind this vigorous development lies a systemic risk driven by climate change.^12^^,^^13^ As the foundation of photovoltaic power generation, the long-term dynamics of surface solar radiation resources are being profoundly impacted by climate change, posing a fundamental challenge to the reliability of energy planning.^14^^,^^15^ Studies based on CMIP6 multi-scenario simulation data indicate that differences in future aerosol emission changes and cloud feedback processes are the primary factors contributing to significant uncertainties in regional surface solar radiation predictions.^16^^,^^17^^,^^18^ Focusing on China’s regional context, Paul Adigun et al. indicate that by the end of the 21st century (2066–2100), China’s solar resource potential will exhibit significant north-south divergence, highly dependent on the emission scenario.^19^ Junhong Guo et al. attribute this divergence to the subtropical regions of southern China being more significantly influenced by monsoon circulation and cloud cover variability, resulting in a higher overall sensitivity of solar resources to climate warming compared to the arid northern regions.^20^ Guangxi, as a quintessential example of subtropical karst topography in southern China, exhibits a fragmented karst peak cluster terrain that further amplifies the disturbance effects of climate-related factors. This results in a more complex response of solar energy resources to climate change within the region, accompanied by heightened prediction uncertainties.^21^^,^^22^^,^^23^ Moreover, Guangxi serves not only as the core corridor for the “West-East Power Transmission” initiative but also as a vital clean energy base underpinning the energy security of the Guangdong-Hong Kong-Macao Greater Bay Area.^24^^,^^25^^,^^26^ Therefore, accurately assessing the spatiotemporal dynamics and migration risks of Guangxi’s future solar energy resources has become a critical prerequisite for implementing its energy strategy.

However, existing research on precise assessments for Guangxi still faces dual challenges posed by its unique complexity and methodological limitations.^27^ Traditional statistical methods struggle to capture the complex spatiotemporal non-stationarity and quantify the interactive mechanisms among factors. For instance, attribution methods relying solely on Pearson correlation coefficients or stepwise regression to quantify linear relationships between meteorological factors and surface solar radiation fail to effectively identify nonlinear coupling effects, multi-factor synergistic interactions, and dynamic correlations under spatiotemporal non-stationarity.^28^^,^^29^^,^^30^ Despite significant progress in cutting-edge research in this field—such as utilizing artificial intelligence models to integrate satellite and ground-based observation data, which has demonstrated exceptional capability in capturing non-stationarity in short-term radiation probability forecasts.^31^^,^^32^^,^^33^ The application of radiant energy feedback/indirect radiation transfer models in meteorological models such as WRF-Solar has improved the physical simulation accuracy of key parameters such as clear-sky irradiance.^34^^,^^35^^,^^36^ However, these approaches still have significant limitations in addressing the challenges of long-term energy planning in Guangxi. The former (AI models) lacks coupling with long-term climate scenarios and has weak physical interpretability, while the latter (high-complexity meteorological models) incurs prohibitive computational costs—making it difficult to support multi-scenario, long-time-series spatiotemporal dynamic analysis and risk quantification.^37^^,^^38^^,^^39^ Therefore, against the backdrop of the strategic decisions for the dual carbon goals, there is an urgent need to establish a comprehensive analytical framework. This framework should be capable of integrating future climate scenarios, incorporating physical mechanisms, resolving complex spatiotemporal dynamics, and directly serving the assessment of energy transition risks.

**Figure 1:**
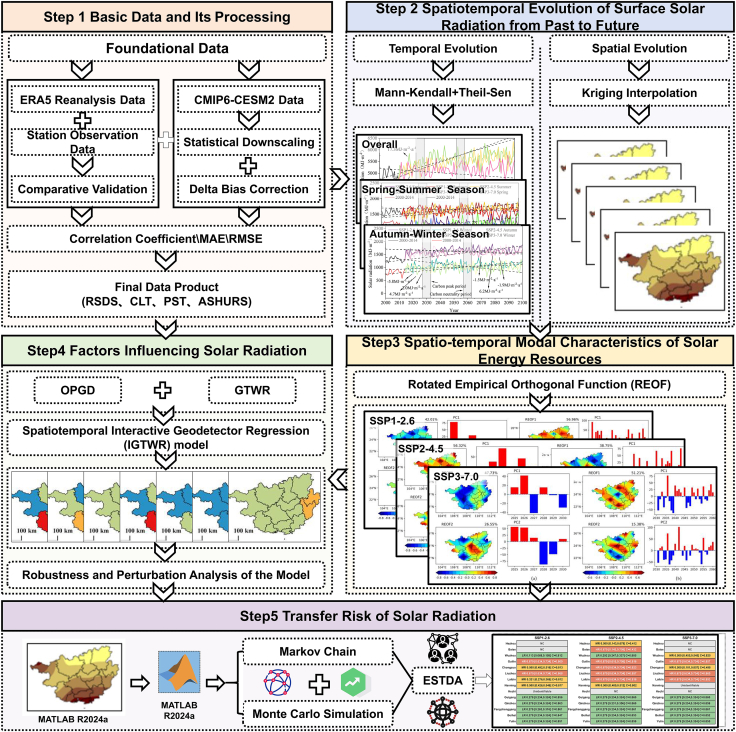
Research technical flowchart (1) The ERA5 reanalysis data and bias-corrected CMIP6 data utilized in Step 1 were validated against recorded data from 44 meteorological stations (1980–2023). The confirmed reliability of these datasets (see Tables S1 and S2 for details) establishes a robust foundation for the subsequent analytical steps depicted in this workflow.

To systematically address the aforementioned challenges—namely the “black-box nature” of AI models, the “high computational cost” of complex meteorological models, and the “static nature” of traditional evaluations—this study constructs a progressive three-tiered analytical framework comprising “model diagnostics, mechanism analysis, and risk assessment” (Figure 1). This framework, driven by CMIP6 future climate scenario data (SSP1-2.6, SSP2-4.5, SSP3-7.0), aims to comprehensively address the core scientific questions: “How do resource patterns evolve temporally and spatially? → What are the underlying driving mechanisms? → What are the risks under multiple scenarios?” through three progressive stages.

Specifically, this study will proceed as follows: First, a combined approach of the Mann-Kendall trend test and Theil-Sen slope estimation will be employed to systematically evaluate the temporal trends in Guangxi’s solar energy resources across the historical period (1970–2024) and the future scenario period (2025–2100). Second, spatial modes will be optimized using the maximum variance rotation method within rotated empirical orthogonal functions (REOF) to identify spatial fractal patterns, key differentiation zones, as well as the spatial clustering and dynamic correlation patterns of high/low-value areas in Guangxi’s solar resource anomalies. Furthermore, this study innovatively coupled the Optimal Parameter Geographic Detector (OPGD) with the Geographically and Temporally Weighted Regression (GTWR) model to construct the spatiotemporal interactive geodetector regression (IGTWR). Through a synergistic design where OPGD explicitly quantifies the interactive explanatory power (q-value) of multi-source climate factors (e.g., cloud cover and aerosols) and GTWR dynamically characterizes their spatiotemporal heterogeneity coefficients, the proposed IGTWR model explicitly analyzes the interactive driving effects of these factors. This approach overcomes the “black box” limitations of implicit fitting in AI models, anchors results to the physical mechanisms of climate factor interactions, and enhances physical interpretability. Finally, the exploratory spatiotemporal data analysis (ESTDA) method—which integrates probabilistic Markov chains with Monte Carlo simulations—is introduced to upgrade static assessments into dynamic risk quantification. This enables the probabilistic characterization of spatiotemporal migration risks for resources under multiple future scenarios, addressing the core challenge of “quantifiable uncertainty” in long-term energy planning. The significance of this study lies not only in revealing the distribution patterns and potential migration risks of future solar resources in Guangxi, providing direct scientific decision-making support for its energy planning, but more importantly, in establishing the IGTWR model and risk quantification paradigm. This framework offers critical modeling and data support for optimizing regional energy decarbonization pathways under high-emission scenarios. Concurrently, this study offers a transferable “scenario-mechanism-risk” integrated assessment methodology and case paradigm for evaluating energy transitions in similar climatic zones, such as Southeast Asia.

## Results

### Spatial and temporal evolution patterns of solar energy resources

**Figure 2:**
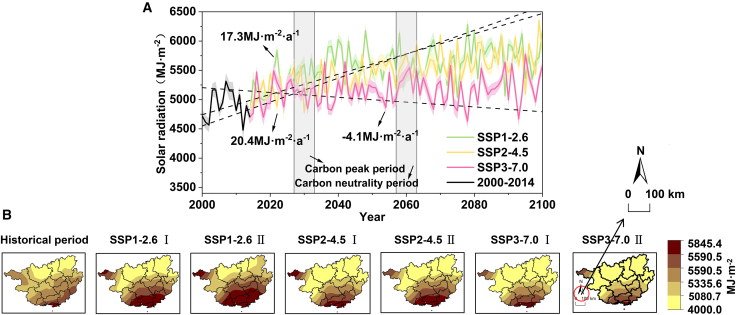
Annual solar radiation trends and distributions in Guangxi (A) Long-term trends of surface solar radiation (SSR; unit: MJ·m^−2^·year^−1^) from 1970 to 2100. The trends are based on the ERA5 reanalysis dataset for 1970–2014 and on the bias-corrected CMIP6-CESM2 dataset under the SSP1-2.6, SSP2-4.5, and SSP3-7.0 scenarios for 2015–2100. Solid lines connect annual data points, illustrating interannual variability, while dashed lines represent the Theil-Sen slope estimates for the long-term trend. All long-term trends are statistically significant (*p < 0.01*; Mann-Kendall test). Data are represented as mean ± SEM (*n* = 14). (B) Spatial distribution of surface solar radiation (unit: MJ·m^−2^·year^−1^). From left to right: Historical mean (1970–2024) based on ERA5 reanalysis, followed by the projected future means for Phase I (carbon peaking, 2025–2050) and Phase II (carbon neutrality, 2051–2100) under the SSP1-2.6, SSP2-4.5, and SSP3-7.0 scenarios, respectively. All future projections are based on the bias-corrected CMIP6-CESM2 dataset. The scale in the map represents 100 km. The base administrative boundary map of Guangxi was downloaded from the Standard Map Service of the Ministry of Natural Resources, China (http://bzdt.ch.mnr.gov.cn/) and was created using ArcGIS 10.8, with no modifications made to the boundaries. This same base map is used for all spatial distribution figures in this study.

Interannual Variability Characteristics: Simulation results under different shared socioeconomic pathways (SSPs) indicate that solar radiation amounts and their spatial distribution exhibit significant dependence on carbon emission scenarios. Specifically, the annual average solar radiation amount is highest under the SSP1-2.6 scenario (5647.78 MJ m^−2^), with a significant increasing rate of 8.99 MJ m^−2^·a^−1^. Spatially, it generally presents a gradient distribution pattern of being higher in the south and lower in the north, with the high-value areas during the carbon neutrality period expanding toward the central region compared to the carbon peaking period. The annual average value under the SSP2-4.5 scenario is 5527.27 MJ m^−2^, which is 120.51 MJ m^−2^ lower than that under the SSP1-2.6 scenario, but its growth rate is slightly faster (9.48 MJ m^−2^·a^−1^). Its spatial distribution pattern is similar to that of SSP1-2.6, except that the scope of high-value areas shrinks toward the south. The annual average value under the SSP3-7.0 scenario is the lowest (5174.23 MJ m^−2^). Although it still maintains growth at a rate of 3.07 MJ m^−2^·a^−1^, its growth trend is significantly weakened compared to the SSP1-2.6 and SSP2-4.5 scenarios, with the growth rate decreasing by approximately 66%. This indicates that high-emission scenarios have begun to inhibit the increase in solar radiation amounts, and their high-value areas have further retreated southward, becoming increasingly concentrated in the southern coastal zones. Notably, annual averages across all scenarios exceed the Grade II standard (GB/T 37526-2019) (Table 1), indicating overall abundant solar resources in the region. Overall, as emission scenarios intensify (SSP1-2.6 to SSP3-7.0), annual average solar radiation decreases progressively. Concurrently, high-value resource-rich zones exhibit a spatial evolution trend of continuous contraction and concentration toward the southern Beibu Gulf coastal areas (Figure 2).

**Table 1:** Classification of solar energy resource (GHR) abundance levels

Global horizontal irradiation grade name^a^	Classification threshold (MJ·m^−2^)^a^
GHR-I (Most abundant)	GHR ≥6300
GHR-II (Very rich)	5040 ≤ GHR <6300
GHR-III (Enrich)	3780 ≤ GHR <5040
GHR-IV (Generally)	GHR <3780

a This classification follows the Chinese national standard GB/T 37526-2019.

**Figure 3:**
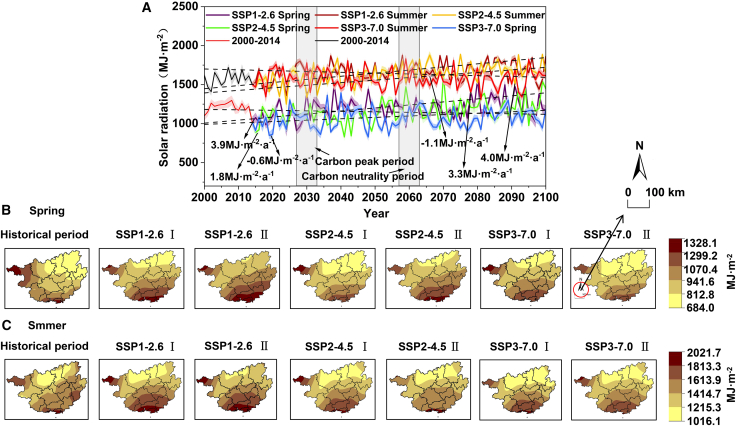
Spring and summer solar radiation trends and distributions in Guangxi (A) Long-term trends of surface solar radiation (SSR; unit: MJ·m^−2^ year^−1^) in spring and summer from 1970 to 2100. The trends are based on the ERA5 reanalysis dataset for 1970–2014 and on the bias-corrected CMIP6-CESM2 dataset under the SSP1-2.6, SSP2-4.5, and SSP3-7.0 scenarios for 2015–2100. Solid lines connect annual data points, illustrating interannual variability, while dashed lines represent the Theil-Sen slope estimates for the long-term trend. All long-term trends are statistically significant (*p < 0.1* for spring and *p < 0.01* for summer; Mann-Kendall test). Data are represented as mean ± SEM (*n* = 14). (B) Spatial distribution of surface solar radiation in spring (unit: MJ·m^−2^·year^−1^). From left to right: Historical mean (1970–2024) based on ERA5 reanalysis, followed by the projected future means for Phase I (carbon peaking, 2025–2050) and Phase II (carbon neutrality, 2051–2100) under the SSP1-2.6, SSP2-4.5, and SSP3-7.0 scenarios, respectively. All future projections are based on the bias-corrected CMIP6-CESM2 dataset. The scale in the map represents 100 km. All maps share the same color scale. (C) Spatial distribution of surface solar radiation in summer (unit: MJ·m^−2^·year^−1^). From left to right: Historical mean (1970–2024) based on ERA5 reanalysis, followed by the projected future means for Phase I (carbon peaking, 2025–2050) and Phase II (carbon neutrality, 2051–2100) under the SSP1-2.6, SSP2-4.5, and SSP3-7.0 scenarios, respectively. All future projections are based on the bias-corrected CMIP6-CESM2 dataset. The scale in the map represents 100 km. All maps share the same color scale.

Seasonal Characteristics: During the “Dual-Carbon” period, solar radiation amounts in summer are generally higher than those in spring (Figure 3). Under the SSP1-2.6 scenario, the annual average solar radiation amount in summer (1674.89 MJ m^−2^, 1.56 MJ m^−2^·a^−1^) is 468.9 MJ m^−2^ higher than that in spring (1205.99 MJ m^−2^, 0.54 MJ m^−2^·a^−1^). Spatially, both exhibit a gradient distribution pattern of being higher in the south and lower in the north, with the core high-value areas stably located in the Beibu Gulf and coastal regions. Among them, the high-value areas in summer extend further toward the central region compared to those in spring, and the overall radiation intensity is higher. By the carbon neutrality period, the high-value areas of solar radiation amounts in both spring and summer have expanded toward the central region compared to the carbon peaking period. Under the SSP2-4.5 scenario, the annual average solar radiation amounts in spring and summer (1166.03 MJ m^−2^ and 1655.91 MJ m^−2^) have decreased by 39.96 MJ m^−2^ and 18.98 MJ m^−2^, respectively, compared to the SSP1-2.6 scenario. Their spatial distribution patterns and temporal variation trends are similar to those of SSP1-2.6, but the change rates have adjusted: the increasing rates in spring and summer have slowed down and risen to 0.85 MJ m^−2^·a^−1^ and 1.90 MJ m^−2^·a^−1^, respectively. Under the SSP3-7.0 scenario, solar radiation amounts in both spring and summer exhibit a slight downward trend (−0.47 MJ m^−2^·a^−1^ and -0.15 MJ m^−2^·a^−1^). Its spatial distribution differs significantly from that of the previous two scenarios, with high-value zones continuing to shift southward and the overall radiation intensity showing a marked decline.

**Figure 4:**
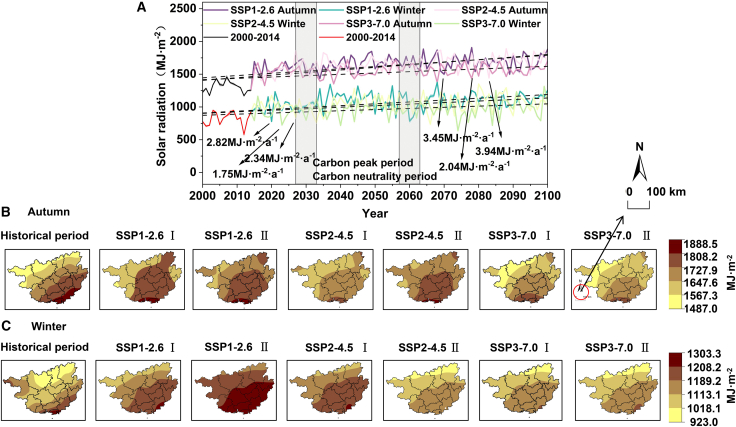
Autumn and winter solar radiation trends and distributions in Guangxi (A) Long-term trends of surface solar radiation (SSR; unit: MJ·m^−2^ year^−1^) in autumn and winter from 1970 to 2100. The trends are based on the ERA5 reanalysis dataset for 1970–2014 and on the bias-corrected CMIP6-CESM2 dataset under the SSP1-2.6, SSP2-4.5, and SSP3-7.0 scenarios for 2015–2100. Solid lines connect annual data points, illustrating interannual variability, while dashed lines represent the Theil-Sen slope estimates for the long-term trend. All long-term trends are statistically significant (*p < 0.01*; Mann-Kendall test). Data are represented as mean ± SEM (*n* = 14). (B) Spatial distribution of surface solar radiation in autumn (unit: MJ·m^−2^·year^−1^). From left to right: Historical mean (1970–2024) based on ERA5 reanalysis, followed by the projected future means for Phase I (carbon peaking, 2025–2050) and Phase II (carbon neutrality, 2051–2100) under the SSP1-2.6, SSP2-4.5, and SSP3-7.0 scenarios, respectively. All future projections are based on the bias-corrected CMIP6-CESM2 dataset. The scale in the map represents 100 km. All maps share the same color scale. (C) Spatial distribution of surface solar radiation in winter (unit: MJ·m^−2^·year^−1^). From left to right: Historical mean (1970–2024) based on ERA5 reanalysis, followed by the projected future means for Phase I (carbon peaking, 2025–2050) and Phase II (carbon neutrality, 2051–2100) under the SSP1-2.6, SSP2-4.5, and SSP3-7.0 scenarios, respectively. All future projections are based on the bias-corrected CMIP6-CESM2 dataset. The scale in the map represents 100 km. All maps share the same color scale.

During the “Dual-Carbon” period, solar radiation amounts in autumn are generally higher than those in winter (Figure 4). Under the SSP1-2.6 scenario, the annual average solar radiation amount in autumn (1674.85 MJ m^−2^) is 577.7 MJ m^−2^ higher than that in winter (1097.15 MJ m^−2^), with both increasing at rates of 3.45 MJ m^−2^·a^−1^ and 2.82 MJ m^−2^·a^−1^, respectively. Spatially, it presents a pattern of being higher in the south and lower in the north, with the high-value area located in south-central Guangxi. Notably, the distribution center of the high-value area of solar radiation in winter is more southerly than that in autumn. Different from spring and summer, the high-value center of solar radiation amount in autumn and winter has significantly shifted southward during the carbon neutrality period compared to the carbon peaking period. Under the SSP2-4.5 scenario, the average solar radiation amounts in autumn and winter (1664.26 MJ m^−2^ and 1045.90 MJ m^−2^) have decreased by 10.59 MJ m^−2^ and 51.25 MJ m^−2^, respectively, compared to the SSP1-2.6 scenario. The increasing rate in autumn has slightly risen to 3.94 MJ m^−2^·a^−1^, while that in winter has decreased to 2.34 MJ m^−2^·a^−1^ (down from the SSP1-2.6 scenario). Spatially, the overall pattern is similar, but the solar radiation amounts have significantly decreased. Under the SSP3-7.0 scenario, the increasing rates of solar radiation amounts in autumn and winter are the lowest among the three scenarios (2.04 MJ m^−2^·a^−1^ and 1.75 MJ m^−2^·a^−1^). Moreover, under this scenario, the high-value areas continue to retreat southward and concentrate in the southern coastal regions, with the overall intensity further declining. This spatial evolution characteristic is highly consistent with that in spring and summer.

### Spatiotemporal modal characteristics of solar energy resources (rotated empirical orthogonal function analysis)

To ensure the statistical significance of the results, we performed a significance test on the REOF modes using the North criterion. The results indicate that the first mode is significantly separated under all scenarios, suggesting it represents an independent and stable dominant spatial distribution pattern. In contrast, the second mode failed the significance test in all cases, implying it may not correspond to an independent physical entity and requiring caution in interpretation. Nevertheless, given its non-negligible average variance contribution rate of approximately 25%, this study still analyzes the second mode to explore potential secondary distribution characteristics, with its physical meaning provided only as a preliminary reference (see Table S3 for detailed results).

#### SSP1-2.6

**Figure 5:**
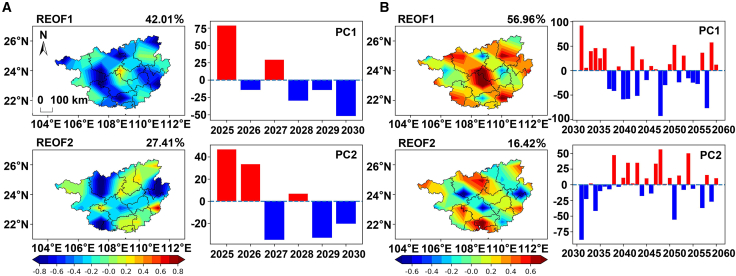
Dominant spatiotemporal modes of solar radiation under SSP1-2.6 (A) Spatial modes (REOF1, REOF2) and their corresponding time series (PC1, PC2) during the carbon peaking period (2025–2030) under the SSP1-2.6 scenario. The scale bar represents 100 km and applies to both (A) and (B). (B) Spatial modes (REOF1, REOF2) and their corresponding time series (PC1, PC2) during the carbon neutrality period (2051–2100) under the SSP1-2.6 scenario. (C) The corresponding leading modes shown in (A) and (B) have been statistically tested and are significant. Detailed results are provided in Table S3.

As shown in Figure 5A, the cumulative variance contribution rate of the first two major spatial modes of solar radiation amount during the carbon peaking period reaches 69.42%, dominating the spatiotemporal variation characteristics of solar radiation amount in this region. The spatial distribution of the first mode (42.01%) presents a differentiated structure consisting of “a small-scale positive anomaly area in western Laibin” and “a large-scale negative anomaly area in other regions” (REOF1 in Figure 5A). Such a spatial structure suggests that when the solar radiation amount in western Laibin is abnormally high (or low), most other prefecture-level cities tend to show the opposite variation trend. The time coefficient of this mode is positive in 2025 and 2027 and shifts to a continuous negative value from 2028 to 2030, with significant anomalies in 2025 and 2030 (|PC| > 2σ) (PC1 in Figure 5A). The spatial distribution of the second mode (27.41%) presents a differentiated structure consisting of “negative anomaly areas in central-southern Guangxi, Hechi, Hezhou, and the southern coastal region” and “positive anomaly areas in other regions” (REOF2 in Figure 5A). Its time coefficient shows that this mode is significantly activated in all years except 2028 and 2030 (|PC| > 2σ).

During the carbon neutrality period, the spatiotemporal variation characteristics of solar radiation amount in this region are also dominated by the first two modes, with a cumulative variance contribution rate of 73.38%. The first mode (56.96%) exhibits a nearly consistent spatial distribution across the entire region, with the positive core area located in Nanning City (REOF1 in Figure 5B). Its time coefficient shows alternating positive and negative oscillations: this mode is moderately activated in 47% of the years (|PC| > σ) and significantly activated in 30% of the years (|PC| > 2σ) (PC1 in Figure 5B). The second mode (16.42%) presents a differentiated structure consisting of “positive anomaly areas along the coast and eastern Hechi” and “negative anomaly areas in the remaining regions” (REOF2 in Figure 5B). The positive core area is located in the southern coastal region, while the negative high-value areas are distributed in Hezhou, southwestern Hechi, and eastern Nanning. Its time coefficient shows that this mode is moderately activated in 20% of the years (|PC| > σ) and significantly activated in 20% of the years (|PC| > 2σ) (PC2 in Figure 5B). Overall, compared with the second mode, the first mode is more representative of the spatiotemporal variation characteristics of solar radiation amount in this region during the carbon neutrality period—both in terms of the cumulative variance contribution rate and the performance of the PC coefficients.

#### SSP2-4.5

**Figure 6:**
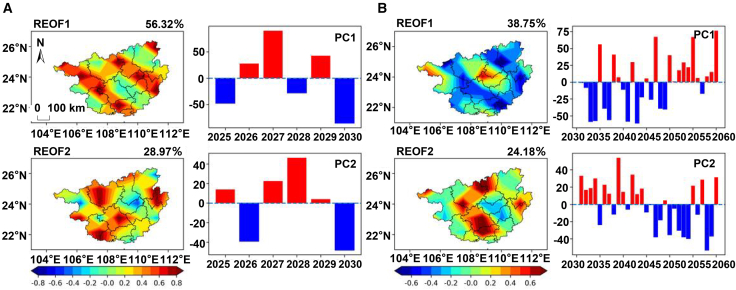
Dominant spatiotemporal modes of solar radiation under SSP2-4.5 (A) Spatial modes (REOF1, REOF2) and their corresponding time series (PC1, PC2) during the carbon peaking period (2025–2030) under the SSP2-4.5 scenario. The scale bar represents 100 km and applies to both (A) and (B). (B) Spatial modes (REOF1, REOF2) and their corresponding time series (PC1, PC2) during the carbon neutrality period (2051–2100) under the SSP2-4.5 scenario. (C) The corresponding leading modes shown in (A) and (B) have been statistically tested and are significant. Detailed results are provided in Table S4.

As shown in Figure 6A, during the carbon peaking period, the cumulative variance contribution rate of the first two major spatial modes of solar radiation amount reaches as high as 85.29%, dominating the spatiotemporal variation characteristics of this region. The first mode (56.32%) exhibits a spatial distribution of “consistent positive values across the entire region,” with the positive core area located in Baise, Wuzhou, Qinzhou, Liuzhou, and the junction of Nanning and Chongzuo (REOF1 in Figure 6A). Its time coefficient shows alternating oscillations. Statistics indicate that this mode is moderately activated in 67% of the years (|PC| > σ) and significantly activated in 33% of the years (|PC| > 2σ) (PC1 in Figure 6A). The second mode (28.97%) presents a differentiated structure consisting of “a negative anomaly area from eastern Laibin to central Baise” and “positive anomaly areas in other regions,” with the positive core area located in Hechi, Fangchenggang, Nanning, Hezhou and other regions (REOF2 in Figure 6A). Its time coefficient also shows alternating oscillations. Statistics indicate that this mode is significantly activated in 50% of the years (|PC| > 2σ), with an activation frequency approximately 1.5 times that of the first mode (33%); however, in the other 50% of the years, its activation intensity is weak (|PC| < 2σ), among which it is moderately activated in 2027 (|PC| > σ) and not effectively activated in 2025 and 2029 (|PC| < σ) (PC2 in Figure 6A). Although the second mode has a higher activation frequency, the first mode remains the most dominant spatiotemporal variation characteristic during this period, thanks to its nearly doubled variance contribution rate (56.32% vs. 28.97%).

During the carbon neutrality period (Figure 6B), the cumulative variance contribution rate of the first two modes of solar radiation is 62.93%. Although it has decreased compared with that during the carbon peaking period (85.29%), the two modes still continue to dominate the spatiotemporal variation characteristics of this region. The first mode (38.75%) presents a differentiated structure consisting of “positive anomaly areas in the north-central, east-central, southern, and western regions” and “negative anomaly areas in the remaining regions” (REOF1 in Figure 6B). Compared with the carbon peaking period, the dominant spatiotemporal mode has undergone a fundamental transformation. The dominant mode with consistent distribution across the entire region has been replaced by a complex spatial differentiation structure, and the activation frequency of the second mode (43%) has exceeded that of the first mode (30%) for the first time. This indicates that the influencing factors tend to be diversified, and the uncertainty of the spatiotemporal distribution of solar radiation amount has increased significantly.

#### SSP3-7.0

**Figure 7:**
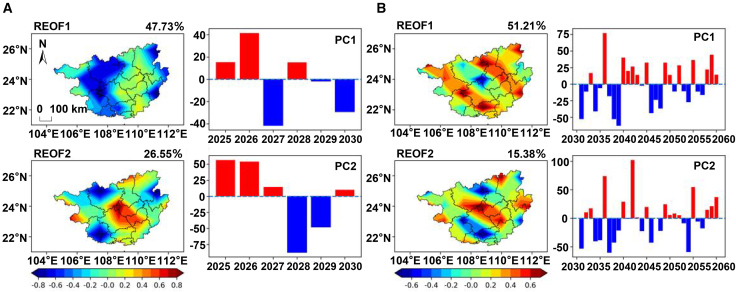
Dominant spatiotemporal modes of solar radiation under SSP3-7.0 (A) Spatial modes (REOF1, REOF2) and their corresponding time series (PC1, PC2) during the carbon peaking period (2025–2030) under the SSP3-7.0 scenario. The scale bar represents 100 km and applies to both (A) and (B). (B) Spatial modes (REOF1, REOF2) and their corresponding time series (PC1, PC2) during the carbon neutrality period (2051–2100) under the SSP3-7.0 scenario. (C) The corresponding leading modes shown in (A) and (B) have been statistically tested and are significant. Detailed results are provided in Table S5.

As shown in Figure 7A, during the carbon peaking period, the cumulative variance contribution rate of the first two major spatial modes of solar radiation amount obtained through REOF decomposition is 74.28%, which dominates the spatiotemporal variation characteristics of this region. The first mode (47.73%) presents a differentiated structure consisting of “negative anomaly areas in the western region and eastern edge” and “near-zero values in the remaining regions,” with the negative core area located in Chongzuo and Hechi (REOF1 in Figure 7A). This mode is moderately activated in both 2026 and 2029 (|PC| > σ) and significantly activated from 2026 to 2028 (|PC| > 2σ) (PC1 in Figure 7A). The second mode (26.55%) presents a differentiated structure consisting of “a negative anomaly area from the southwest to Hechi and Hezhou” and “positive anomaly areas in the remaining regions,” with the positive high-value areas concentrated in the central region (REOF2 in Figure 7A). The time coefficient shows that this mode is activated in 67% of the years (|PC| > σ) and significantly activated in 2025 and 2028 (PC2 in Figure 7A). Compared with the first mode, the variance contribution rate of the second mode is 21.18 percentage points lower, and its frequency of significant activation is also relatively lower.

During the carbon neutrality period (Figure 7B), the cumulative variance contribution rate of the first two spatial modes of solar radiation amount is 74.28%, which continues to dominate the spatiotemporal variation characteristics of solar radiation amount in this region. The first mode (51.21%) presents a differentiated structure consisting of “a small-scale negative anomaly area in western Laibin” and “positive anomaly areas in other prefecture-level cities” (REOF1 in Figure 7B). Its time coefficient shows that this mode is moderately activated in 27% of the years (|PC| > σ) and significantly activated in 33% of the years (|PC| > 2σ) (PC1 in Figure 7B). The second mode (15.38%) presents a differentiated structure consisting of “a negative anomaly area from the southwest to Hechi” and “positive anomaly areas in the remaining regions” (REOF2 in Figure 7B). Its time coefficient indicates that this mode is moderately activated in 20% of the years (|PC| > σ) and significantly activated in 20% of the years (|PC| > 2σ) (PC2 in Figure 7B).

### Driving mechanism of dynamic changes in solar energy resources based on the spatiotemporal interactive geodetector regression model

#### Performance validation

To verify that the performance of the IGTWR model is superior to that of the ordinary least squares (OLS), geographically weighted regression (GWR), and geographically and temporally weighted regression (GTWR) models, this study selects data from the carbon peaking and carbon neutrality periods under the SSP1-2.6 scenario for comparative analysis. First, the optimal parameter geographical detector (OPGD) is used to screen out influencing factors that pass the significance test and have large qv values (Table 2). Subsequently, a multicollinearity test was conducted on the 6 selected driving factors and 2 groups of factor interaction terms. The results show that the variance inflation factor (VIF) of all variables is below the threshold, indicating no multicollinearity issue (VIF <7.5) (Table 3). Finally, the data were divided into two groups based on whether factor interactions are considered, and OLS, GWR, GTWR, and IGTWR modeling analyses were performed, respectively. This comparative experiment aims to quantitatively evaluate the individual and combined contributions of introducing spatiotemporal nonstationarity and factor interaction effects to improving the explanatory power of the model.

**Table 2:** Results of influencing factor screening by the OPGD

Group 1 (Carbon Peaking Period)	q_v_^a^	*p* < 0.05	Group 2 (Carbon Neutrality Period)	q_v_^a^	*p* < 0.05
Near-Surface Air Temperature (X4)	0.917	Yes	Surface Air Pressure (X3)	0.870	Yes
Ambient Aerosol Optical Depth at 870 nm (X7)	0.851	Yes	Near-Surface Air Temperature (X4)	0.857	Yes
Elevation (X8)	0.832	Yes	Elevation (X8)	0.828	Yes
Interaction Term of Surface Air Pressure and Ambient Aerosol Optical Thickness at 550 nm (X3X6)	0.874	Yes	Interaction Term of Total Cloud Cover Percentage and Ambient Aerosol Optical Thickness at 440 nm (X1X5)	0.929	Yes

a The q_v_ represents the explanatory power of the driver, with a range of [0, 1]. All listed drivers are statistically significant at the 0.05 level (determined by the Monte Carlo randomization test).

**Table 3:** Multicollinearity test results

Group 1 (Carbon peaking period)	VIF^a^	Group 2 (Carbon neutrality period)	VIF^a^
Near-Surface Air Temperature (X4)	7.355	Surface Air Pressure (X3)	5.699
Ambient Aerosol Optical Depth at 870 nm (X7)	4.658	Near-Surface Air Temperature (X4)	5.126
Elevation (X8)	4.699	Elevation (X8)	5.608
Interaction Term of Surface Air Pressure and Ambient Aerosol Optical Thickness at 550 nm (X3X6)	1.280	Interaction Term of Total Cloud Cover Percentage and Ambient Aerosol Optical Thickness at 440 nm (X1X5)	1.005

a Variance inflation factor (VIF). All VIF values are below the common threshold of 10, indicating no severe multicollinearity among the drivers in each period.

As shown in Table 4, the model goodness-of-fit was first evaluated based on the coefficient of determination (R^2^ and adjusted R^2^): without introducing factor interaction terms, the adjusted R^2^ of the GTWR model reached 0.996 and 0.740 during the carbon peaking and carbon neutrality periods, respectively, which was significantly superior to those of the OLS model (0.906 and 0.653) and the GWR model (0.959 and 0.704). This confirms the importance of considering spatiotemporal heterogeneity in modeling. After introducing factor interaction effects, the fitting performance of all models was systematically improved, with the increase in adjusted R^2^ exceeding 2.08%. This indicates that the interaction effects between factors are a non-negligible influencing mechanism (Tables 4 and 5). Having established the superior explanatory power of the IGTWR model, we further confirmed the statistical significance and robustness of its estimation results through a Bayesian analysis (Tables S6 and S7). Therefore, in the subsequent analysis, we will conduct an in-depth analysis of the driving factors based on the estimation results of the IGTWR model.

**Table 4:** Results of model comparison experiment (group 1)

Measure\Model^a^	OLS (W/O^d^)	GWR (W/O^d^)	GTWR (W/O^d^)	OLS (W/e)	GWR (W/e)	IGTWR (W/e)
R^2^^b^	0.862	0.934	0.962	0.910	0.961	0.997
AdjustedR^2^^b^	0.857	0.932	0.961	0.906	0.959	0.996
AICc^c^	82.735	62.783	59.178	49.053	39.815	25.136

a Group 1 refers to the data from the carbon peaking period (2025–2050) under the SSP1-2.6 scenario.

b R^2^ and Adjusted R^2^ represent the goodness-of-fit, with values closer to 1 indicating a better fit.

c Akaike Information Criterion corrected for small sample size (AICc). Lower values indicate a better model with a preferred balance of goodness-of-fit and simplicity.

d W/O indicates without factor interactions.

e W/ indicates with factor interactions.

**Table 5:** Results of model comparison experiment (group 2)

Measure\Model^a^	OLS (W/O^d^)	GWR (W/O^d^)	GTWR (W/O^d^)	OLS (W/e)	GWR (W/e)	IGTWR (W/e)
R^2^^b^	0.651	0.694	0.742	0.656	0.707	0.881
AdjustedR^2^^b^	0.648	0.691	0.740	0.653	0.704	0.880
AICc^c^	760.159	731.707	710.785	755.758	716.092	465.741

a Group 2 refers to the data from the carbon neutrality period (2051–2100) under the SSP1-2.6 scenario.

b R^2^ and adjusted R^2^ represent the goodness-of-fit, with values closer to 1 indicating a better fit.

c Akaike Information Criterion corrected for small sample size (AICc). Lower values indicate a better model with a preferred balance of goodness-of-fit and simplicity.

d W/O indicates without factor interactions.

e W/ indicates with factor interactions.

#### Results analysis

**Figure 8:**
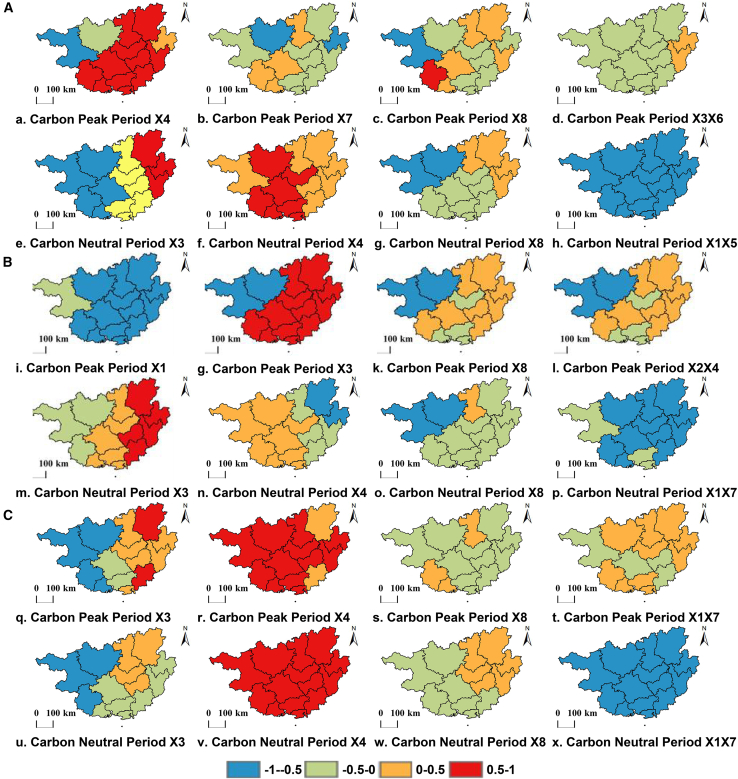
IGTWR regression coefficients for major drivers (SSPx) (A) Spatial distribution of regression coefficients for key drivers during the carbon peaking (a-d) and carbon neutrality (e-h) periods under the SSP1-2.6 scenario. (B) Spatial distribution of regression coefficients for key drivers during the carbon peaking (i-l) and carbon neutrality (m-p) periods under the SSP2-4.5 scenario. (C) Spatial distribution of regression coefficients for key drivers during the carbon peaking (q-t) and carbon neutrality (u-x) periods under the SSP3-7.0 scenario. Scale bars in all maps represent 100 km.

Under the SSP1-2.6 scenario, the dominant drivers during the carbon peaking period include X4 (Near-surface air temperature, Figure 8A), X7 (Ambient aerosol optical depth at 870 nm, Figure 8B), X8 (Elevation, Figure 8C), and the interaction term X3X6 (Surface air pressure × aerosol optical thickness at 550 nm, Figure 8D).X4 exerts a consistently positive effect across the entire study area, suggesting that warming generally enhances surface solar radiation. X3X6 exhibits a uniformly negative effect across the entire region, indicating that their synergy significantly weakens radiation. X7 and X8 are highly heterogeneous, indicating that their effects depend strongly on local conditions. By the carbon neutrality period, the dominant drivers change significantly, shifting to X3 (surface air pressure, Figure 8E), X4 (near-surface air temperature, Figure 8F), X8 (elevation, Figure 8G), and the interaction term X1X5 (Total cloud cover percentage × ambient aerosol optical thickness at 440 nm, Figure 8H). Notably, the regression coefficient of the interaction term X1X5 is significantly negative across the entire region, indicating that it exerts a stable synergistic weakening effect on solar radiation amount during this period. However, the regression coefficients of other major factors show significant regional differences, reflecting greater local dependency.

Under the SSP2-4.5 scenario, solar radiation during the carbon peaking period is primarily driven by X1 (Total Cloud Cover Percentage, Figure 8I), X3 (surface air pressure, Figure 8J), X8 (Elevation, Figure 8K), and the interaction term X2X4 (humidity × temperature interaction, Figure 8L). During this period, X1 is the dominant control, exerting a stable weakening effect on radiation across the region, while the impacts of other factors show clear regional dependence, reflecting complex local regulatory processes. By the carbon neutrality period, the drivers shift to X3 (surface air pressure, Figure 8M), X4 (near-surface air temperature, Figure 8N), X8 (elevation, Figure 8O), and the interaction term X1X7 (total cloud cover × coarse-mode aerosol optical depth interaction, Figure 8P). The X1X7 interaction exhibits a consistently strong negative effect across the entire study area (regression coefficient < −0.5), indicating a stable inhibitory synergy between cloud cover and coarse-mode aerosols on radiation. In contrast, all other factors show distinct spatial heterogeneity, with both the direction and magnitude of their effects remaining strongly region-dependent.

Under the SSP3-7.0 scenario, the dominant drivers during the carbon peaking period are X3 (surface air pressure, Figure 8Q), X4 (near-surface air humidity, Figure 8R), X8 (elevation, Figure 8S), and the interaction term X1X7 (total cloud cover percentage × ambient aerosol optical thickness at 440 nm, Figure 8T). With the exception of X4, all other drivers show strong spatiotemporal heterogeneity, with both the magnitude and sign of their regression coefficients heavily influenced by local conditions. The X1X7 interaction exerts a moderate inhibitory effect during this period but lacks a spatially consistent pattern across the region. By the carbon neutrality period, the drivers shift to X3 (Surface air pressure, Figure 8U), X4 (Near-surface air temperature, Figure 8V), X8 (Elevation, Figure 8W), and the interaction term X1X7 (total cloud cover percentage × ambient aerosol optical depth at 870 nm, Figure 8X). Similar to the SSP2-4.5 scenario, the X1X7 interaction exhibits a significant and spatially consistent inhibitory effect across the entire region, with its impact strength further enhanced. This underscores the key regulatory role of aerosol-cloud interactions in the radiation balance under high-emission scenarios. The remaining factors continue to exhibit pronounced regional heterogeneity, highlighting the importance of local processes.

### Risk assessment of solar energy resource migration

**Figure 9:**
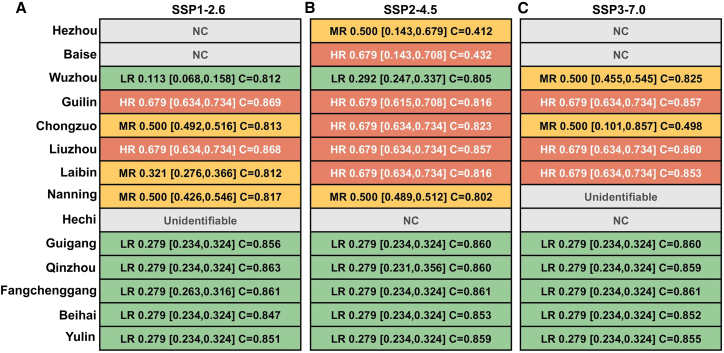
The migration risk of solar energy resources in Guangxi (A) Risk assessment under the SSP1-2.6 scenario. (B) Risk assessment under the SSP2-4.5 scenario. (C) Risk assessment under the SSP3-7.0 scenario. For each city and scenario, the table presents the risk level and a detailed probabilistic assessment. Risk levels are defined as: NC (no change), LR (low risk, <0.3), MR (medium risk, 0.3–0.6), and HR (high risk, >0.6). Data points that were unreasonable due to model constraints or input quality are marked as “Unidentifiable.” These threshold values (0.3, 0.6) were predefined as fixed criteria within the risk assessment framework prior to model execution to ensure an objective and consistent classification scheme. Each cell’s content follows the format: level symbol probability [confidence lower, confidence upper] C = robustness score, where probability is the central estimate of the migration risk index; [confidence lower, confidence upper] represents the 95% confidence interval derived from the Bootstrap Monte Carlo simulation (see STAR Methods); and C = robustness score indicates the reliability of the prediction, calculated based on migration rationality and transition matrix stability, with higher scores representing greater robustness. The risk probabilities and confidence intervals are derived from a probabilistic assessment framework that models LISA spatiotemporal state sequences as a Markov process, with uncertainty quantified using parametric Bootstrap resampling (*n* = 300 replicates). See also the STAR Methods section “ESTDA Based on Markov Chain and Monte Carlo Methods” for full methodological details.

Having analyzed the dynamics of solar resources in Guangxi, assessing their future stability under climate change is crucial for energy security and strategic planning. Therefore, this study quantifies the risk of its resource migration by introducing a probabilistic risk assessment framework integrating Markov chains and Monte Carlo simulations (Figure 9).

The experimental results show that under the SSP1-2.6 scenario, Guilin and Liuzhou are identified as high-risk areas (HR), both with a migration probability of 0.679 and high model robustness (C > 0.86). Nanning, Laibin, and Chongzuo are identified as medium-risk areas (MR), with migration probabilities of 0.500, 0.321, and 0.500, respectively. Most cities, such as Wuzhou, Guigang, Qinzhou, Fangchenggang, Beihai, and Yulin, are identified as low-risk (LR) areas. These classifications are also supported by high model robustness (C > 0.8). Hezhou and Baise are categorized as “no change” (NC). Hechi is classified as “indeterminate” due to a non-monotonic probability trend across scenarios (i.e., failing the condition SSP1-2.6 < SSP2-4.5 < SSP3-7.0). Although some similar cities, such as Baise, under the SSP2-4.5 scenario also do not satisfy the above condition, their results are retained to faithfully represent the uncertainties inherent in complex system responses, given their low confidence intervals and reliability scores, which indicate significant uncertainty.

Under the SSP2-4.5 scenario, the resource risk pattern undergoes a significant change. The number of high-risk cities increases to include Baise, Guilin, Chongzuo, Liuzhou, and Laibin. Among them, Guilin, Chongzuo, Liuzhou, and Laibin exhibit high robustness (C-values all greater than 0.81). Hezhou transitions from “no change” (NC) to medium-risk (MR), but the assessments for Hezhou and Baise are associated with wide confidence intervals, indicating substantial uncertainty. Hechi remains “no change” (NC), while Fangchenggang, Guigang, Qinzhou, Beihai, and Yulin maintain low-risk (LR) status with generally high robustness. Under the SSP3-7.0 scenario, resource risk intensifies further. Guilin, Liuzhou, and Laibin are identified as high-risk (HR) areas with high robustness (C-values all greater than 0.85). Wuzhou shifts to a medium-risk area (MR), while Chongzuo transitions from high-risk (HR) to medium-risk (MR). However, the assessment for Chongzuo has low robustness (C = 0.498) and an extremely wide confidence interval [0.101, 0.857], rendering it highly uncertain. The risk classification for Nanning is inconclusive (indeterminate). Guigang, Qinzhou, Fangchenggang, Beihai, and Yulin continue to maintain a low-risk (LR) status with high robustness. Hezhou, Baise, and Hechi show no changes (NCs).

In summary, the migration risk of solar energy resources in Guangxi exhibits a predominant spatial pattern of “high inland and low coastal,” and the risk level increases significantly with the intensification of emission scenarios. Although there is uncertainty in the assessment of some regions, the identification results of major high-risk and low-risk cities are robust and reliable. These assessment results provide a basis for formulating differentiated regional energy strategies under future climate change.

## Discussion

Although this study systematically analyzed the dynamic distribution patterns and driving mechanisms of solar energy resources in Guangxi based on the MK-TS test, REOF analysis, and IGTWR model, the significant patterns identified in the conclusions and the intrinsic connections between various conclusions have not been fully elaborated. Meanwhile, there is a lack of horizontal comparison with patterns in other regions, which makes the integrity and universality of the research need to be further deepened. Therefore, this chapter will address the aforementioned shortcomings by focusing on: (1) the significant patterns identified in the conclusions and their underlying mechanisms, (2) the intrinsic connections and mutual validation among the conclusions, (3) the regional specificity and universality of the findings (horizontal comparisons), and (4) the implications of these comprehensive findings for future energy planning, particularly in contexts of uncertainty.

### The non-linear response of solar radiation to emission pathways

In the section titled “Spatial and Temporal Evolution Patterns of Solar Energy Resources,” we analyzed the interannual and seasonal spatiotemporal variation patterns of solar energy resources in Guangxi. It is noteworthy that the TS estimated slope values for interannual and seasonal variations under all scenarios follow the order SSP2-4.5 > SSP1-2.6 > SSP3-7.0 (except in winter). Although this order does not intuitively follow the intensity gradient of emission pathways, there is a reasonable physical mechanism behind it. Specifically, under the intermediate SSP2-4.5 scenario, the emission reduction rate of scattering particles such as aerosols in the atmosphere may be faster than the growth of greenhouse gas concentrations. This asynchronous emission reduction leads to an atmospheric effect similar to “purification” (i.e., a rapid decrease in aerosol optical depth). As a result, during the transitional stage where the greenhouse effect has not yet fully dominated, the solar radiation reaching the surface (especially the direct radiation component) shows a relatively significant enhancement. In the low-carbon SSP1-2.6 scenario, the coordinated and rapid emission reduction of both greenhouse gases and aerosols weakens the intensity of this “purification effect.” In contrast, under the high-carbon SSP3-7.0 scenario, the sharp rise in greenhouse gas concentrations dominates. The positive feedback processes it triggers—such as increased water vapor, changes in cloud cover, and secondary aerosol formation—not only completely offset any potential radiation enhancement but also significantly inhibit and reverse the growth trend of solar radiation. Therefore, the aforementioned seemingly anomalous pattern essentially reflects the result of the nonlinear interaction between different forcing factors under different emission pathways.

### Converging evidence from rotated empirical orthogonal functions modes and drivers

In addition, there exists a profound intrinsic connection and mutual verification relationship between the conclusions of the “Spatio-temporal modal characteristics of solar energy resources (REOF Analysis)” section and those of the “Analysis of Driving Factors Based on the IGTWR Model” section. Specifically, the REOF analysis identified the key modes (i.e., “distribution patterns”) dominating the spatial differentiation of solar energy resources in Guangxi under different scenarios and periods; this precisely provides an intuitive “outcome” for the driving mechanisms revealed by the IGTWR model. Conversely, the spatiotemporal distribution maps of regression coefficients in the IGTWR driver analysis (Figure 8) clearly depict the spatial pattern of the influence of each driving factor (i.e., the “causes of action”). By systematically comparing the similarity between the spatial patterns of REOF modes and the spatial distributions of regression coefficients of key driving factors, we can establish a bridge from “phenomena” to “causes.” For example, when the high-value area of a certain mode highly coincides with the strong negative impact area of a driving factor (such as aerosol optical depth), it strongly confirms that this factor is the dominant driving force for the formation of this distribution mode. This mutual verification between “phenomena” and “mechanisms” in the spatiotemporal dimension not only enhances the reliability of each conclusion but also improves the integrity and persuasiveness of this study’s explanation of the distribution patterns and driving mechanisms of solar energy resources in Guangxi as a whole.

Combining the results of the two sections, it can be concluded that under the SSP1-2.6 (low-carbon) scenario, the driving mechanism evolves from terrain dominance (during the carbon peaking period, elevation X8) to atmospheric environment dominance (during the carbon neutrality period, the interaction term of cloud cover and aerosols X1X5). This reflects the profound impact of reduced anthropogenic emissions on the local climate and environment under the low-carbon pathway. Under the SSP2-4.5 (intermediate) scenario, cloud cover (X1) continues to play a core role throughout the carbon peaking period, indicating the stable role of natural climate factors under the baseline pathway. Due to the limitation of data resolution in attribution analysis, the driving mechanism during the carbon neutrality period cannot be well identified. Under the SSP3-7.0 (high-carbon) scenario, the driving mechanism is more complex, evolving from thermodynamic factor dominance (during the carbon peaking period, atmospheric pressure X3) to composite driving (during the carbon neutrality period, temperature and the interaction term of cloud cover and coarse-mode aerosols X1X7). This implies the enhanced nonlinear response of the climate system and the synergistic effect of multiple factors under the high-forcing scenario.

### Regional specificity and universality: A comparative perspective

The patterns revealed in this study show clear commonalities and characteristics when compared with those in different geographical and climatic regions at home and abroad. The “purification effect” resulting from aerosol emission reduction under the SSP2-4.5 pathway is not only consistent with the mechanism behind the “from gray to bright” trend observed in domestic industrial regions such as North China and the Yangtze River Delta but also corroborates the solar radiation recovery phenomenon witnessed in Europe and the United States during the post-industrialization period, driven by the implementation of clean air policies. Together, they validate the cross-regional universal law that anthropogenic aerosol changes exert a key regulatory role on surface solar radiation.^40^^,^^41^^,^^42^^,^^43^^,^^44^ Meanwhile, Guangxi, as a subtropical humid hilly region, exhibits a unique dynamic response pattern. It differs from the Qinghai-Tibet Plateau, where terrain plays a stably dominant role, and the arid northwest region is dominated by sand and dust. It also stands in sharp contrast to the Southeast Asian monsoon region, which will still face pressure from increasing aerosol emissions in the future. Under the low-carbon pathway, Guangxi’s driving mechanism shows a clear trajectory of evolution from terrain dominance to cloud-aerosol interaction. This characteristic indicates that deep emission reduction is reconstructing the local climate and environment of such subtropical hilly regions, shifting the dominant factor of their solar energy resources from relatively static geographical elements to dynamic atmospheric processes. This comparison indicates that the conclusions of this study not only corroborate the cross-regional universality of aerosol impacts but also reveal the unique dynamic response mechanism of subtropical hilly regions under future climate pathways, providing new scientific insights for energy and climate risk assessment in similar regions worldwide.

### Implications for energy planning under uncertainty

After clarifying the dynamic patterns and driving mechanisms of solar energy resources in Guangxi, how to intuitively present their future evolution pathways has become a key link supporting scientific decision-making. The reliability of the ESTDA model integrating Markov chains and Monte Carlo simulation has been verified in the “Risk Assessment of Solar Energy Resource Migration” section. To systematically assess the reliability of the IGTWR model, we conduct validation from two dimensions: statistical robustness and sensitivity to data perturbations. As the emission scenario upgrades from SSP1-2.6 to SSP3-7.0, the scope of high-risk areas expands significantly, and resource instability intensifies.

First, there is a profound causal relationship between the risk landscape and the driving mechanisms of resource dynamics. As elaborated earlier in this Discussion, the SSP2-4.5 scenario exhibits the largest increase in solar energy resources due to its “purification effect,” while the SSP3-7.0 scenario suppresses radiation growth due to the strong greenhouse effect. More importantly, we found that the complexity of the driving mechanisms determines the instability of regional resources. The assessment results in this section indicate that it is precisely in the regions with the most drastic resource changes (such as Guilin, Liuzhou, and Laibin) that the migration risks are also the highest. This is no coincidence. Because the driving mechanisms in these regions (e.g., under the SSP3-7.0 scenario) have transformed into complex multi-factor synergistic driving. Such nonlinear responses inherently imply high uncertainty and instability in system outputs, thereby amplifying the long-term uncertainty faced by energy infrastructure planning. Therefore, the inherent source of the persistent high-risk status of regions such as Guilin and Liuzhou lies precisely in this nonlinear response of the climate system.

Secondly, the “uncertainty” in the assessment results itself holds significant scientific value. We interpret uncertainty into two categories: first, for cities such as Liuzhou and Guilin, their risk assessments feature high robustness (high C-values) and narrow confidence intervals. This provides a reliable basis for formulating firm long-term energy strategies, such as exercising prudence in investing in large-scale photovoltaic bases in high-risk areas. Second, for cities such as Chongzuo under the SSP3-7.0 scenario (with extremely wide confidence intervals) or Nanning, where data are “unidentifiable,” their high uncertainty precisely reveals the complexity and unpredictability of the climate-environmental system response in these regions under future high-forcing scenarios. This alerts decision-makers that flexible and resilient adaptation strategies should be prioritized in these areas—such as developing distributed energy storage and building diversified hybrid energy systems—to enhance resilience in coping with unknown risks.

### Limitations of the study

Despite the preliminary progress achieved in this study regarding the assessment of the dynamics and migration risks of solar energy resources in Guangxi, further advancements are still needed in the following three dimensions: first, shifting from single-energy assessment to “wind-solar-storage” synergistic assessment to systematically reveal the potential of multi-energy complementarity in enhancing the climate resilience of regional energy systems^45^^,^^46^; second, moving from traditional methods to data-driven approaches, breaking through scale limitations by integrating high-resolution CMIP6 data with machine learning to accurately quantify extreme climate risks and analyze their physical mechanisms; third, extending from physical risk assessment to comprehensive decision support, conducting socio-economic cost-benefit analysis oriented toward the “dual carbon” goals to provide a scientific basis for formulating regional energy strategies that balance climate resilience and economic benefits.

## Resource availability

### Lead contact

Requests for further information and resources should be directed to and will be fulfilled by the lead contact, Xiangling Tang (txling@glut.edu.cn).

### Materials availability

This study did not generate new datasets.

### Data and code availability

• Processed solar radiation data for Guangxi are available from the lead contact upon reasonable request. Raw meteorological station data are restricted due to confidentiality agreements.
• All original code is available in this article’s supplemental information.
• Any additional information required to reanalyze the data reported in this article is available from the lead contact upon request.

## Acknowledgments

This work was supported by the 10.13039/501100013142Key Research and Development Project of the 10.13039/501100017693Guilin Science and Technology Bureau (Grant No. 20230111-1). The authors are sincerely grateful for this support. We also deeply thank the editors and reviewers for their valuable comments, which have greatly improved the quality of this article.

## Author contributions

Yisong Han conceived the study framework and drafted the article. Xiangling Tang supervised its revision. Wei Li and Siyi Hu were responsible for polishing the text. All authors reviewed the article.

## Declaration of interests

The authors declare no competing interests.

## STAR★Methods

### Key resources table

**Table:** 

REAGENT or RESOURCE	SOURCE	IDENTIFIER
**Deposited data**		

Meteorological Station Data (Guangxi)	National Meteorological Stations (China) Coverage: 1980-2023; Variables: rsds, clt, ps, tas, hurs (daily)	N/A
ERA5 Reanalysis Data	ECMWF Coverage: 1941-2024; Variables: rsds, clt, ps, tas, hurs (daily)	CDS: https://doi.org/10.24381/cds.adbb2d47
CMIP6 CESM2 Model Output	WCRP CMIP6 Coverage: 1941-2024; Variables: rsds, clt, ps, tas, hurs (monthly); Member: r1i1p1f1	GMD: https://doi.org/10.5194/gmd-13-4823-2020
CMIP6 IPSL-CM6A-LR Model Output	WCRP CMIP6 Coverage: 2025-2100; Variables: od440aer, od550aer, od870aer (monthly); Member: r11i1p1f1	GMD: https://doi.org/10.5194/gmd-13-2015-2020

**Software and algorithms**		

MATLAB R2024a	MathWorks	https://www.mathworks.com/downloads/
PyCharm 2024.2.6	JetBrains	https://www.jetbrains.com/pycharm/download/other.html
ArcMap 10.8.2	Environmental Systems Research Institute	https://www.arcgis.com/downloads/
RStudio	Posit Software, PBC	https://posit.co/download/rstudio-desktop/
GeoDa	CSDS	https://geodacenter.github.io/
Code to reproduce the results of this study	Authors (This study)	Data S1–S6 (See data and code availability)

### Method details

#### Data validation and preprocessing

The data used for data preprocessing and bias correction are mainly raster data (multi-year averages of each variable from 1980 to 2023), with the original data as shown in the key resources table. Due to the inherent limitations of CMIP6 models in their parameterization schemes and spatial resolution, their simulation results often contain systematic biases that require correction using observational data. However, the obtained meteorological station data in Guangxi are relatively sparse in spatial distribution, making it difficult to directly serve as a reliable benchmark for downscaling and bias correction. To address this issue, this study designed a progressive data processing and validation workflow. Specifically, first, using the latitude and longitude coordinates of the obtained station observational data, the corresponding data from the ERA5 reanalysis data were extracted based on the “Extract Multi Values to Points” tool in ArcMap 10.8.2 for verification and evaluation to confirm that it meets the conditions for serving as a highly reliable benchmark dataset. Subsequently, taking the validated ERA5 data as the benchmark, a method combining statistical downscaling based on k-nearest neighbor search and Delta bias correction was adopted to correct and downscale the CMIP6 data to a high-resolution grid of 0.25°×0.25°. Building on this, kriging interpolation was further used for post-processing of the results in ArcMap 10.8.2 to optimize their spatial continuity. Finally, to systematically assess the quality of the final data product, the corrected CMIP6 data were compared and validated against the same set of station observations.

#### Temporal evolution characteristics of solar energy resources

Based on the combined method of Mann-Kendall (MK) trend test and Theil-Sen (TS) trend estimation, this study divided the processed annual total and seasonal total solar radiation amount data from 2000 to 2100 into three scenarios (SSP1-2.6, SSP2-4.5, SSP3-7.0) for temporal trend analysis. Statistical significance was evaluated using the Mann-Kendall test, with significance levels set at p < 0.01, p < 0.05, and p < 0.1.

#### Spatio-temporal modal characteristics of solar energy resources

Although the combined MK-TS method can effectively assess the temporal evolution trends of regional solar energy resources, its results struggle to intuitively capture the spatial heterogeneity and regional differentiation patterns of solar energy resources under Guangxi’s complex terrain. To address this limitation, we further introduced the Rotated Empirical Orthogonal Function (REOF) to analyze the spatiotemporal data of solar radiation amount in Guangxi, aiming to identify the core regions of radiation change at different spatial scales.

#### Driving mechanism of dynamic changes in solar energy resources based on the IGTWR model

To reveal the intrinsic correlation between the dominant modes of solar radiation and local climate factors in the study area and accurately capture the spatiotemporal non-stationarity and interaction effects of the impacts of driving factors, this study constructed the Interactive Geographically and Temporally Weighted Regression (IGTWR) model. By introducing significant interaction terms screened by the Optimal Parameter-based Geographical Detector (OPGD) into the traditional Geographically and Temporally Weighted Regression (GTWR) framework, this model enhances its explanatory power and prediction accuracy.

Existing studies have indicated that aerosol emissions, increased atmospheric water-holding capacity caused by global warming, and internal variability factors of the climate system may all be important reasons for the reduction of surface solar radiation. Based on this, this study takes solar radiation as the dependent variable and selects the following core physical variables as driving factors for systematic modeling analysis.

The selection rationale for these core physical variables is as follows:

Cloud Cover (X1) and Relative Humidity (X2): These are the most direct atmospheric parameters influencing the scattering and absorption processes of solar radiation.

Surface Air Pressure (X3) and Near-Surface Air Temperature (X4): These variables characterize the background atmospheric state and thermal conditions, which affect atmospheric transmittance and radiative balance.

Aerosol Optical Depth at 440 nm, 550 nm, and 870 nm (X5, X6, X7): This represents the attenuating effect of aerosols (via absorption and scattering) on radiation, a key anthropogenic and natural factor in radiation dimming. The three wavelengths provide a comprehensive characterization of aerosol properties.

Elevation (X8): As an intrinsic geographic factor of the underlying surface, elevation modulates solar radiation by influencing atmospheric column thickness and local circulation patterns.

The original data sources, spatiotemporal coverage, resolutions, and access identifiers for all variables listed above are fully documented in the key resources table.

In addition, to further explore the differences in driving mechanisms under different climate policy backgrounds, this study sets up multiple modeling scenarios based on the combination of Shared Socioeconomic Pathways (SSPs) and the periods of China’s “dual carbon” (carbon peaking and carbon neutrality) goals. Separate IGTWR models are constructed for each group to systematically reveal the spatiotemporal heterogeneity and interaction mechanisms of solar radiation-influencing factors in different development paths and stages. The Optimal Parameter-based Geographical Detector (OPGD) was implemented by running code in RStudio. Both the intermediate validation process and the final construction and calculation workflow of the IGTWR model were completed by downloading the GTWR plugin into ArcMap 10.8.2.

#### Assessment of solar energy resource migration risks based on the ESTDA model

Although the MK-TS test, REOF analysis, and IGTWR model have revealed the spatiotemporal evolution trends, key anomalous modes, and driving factors of solar energy resources in Guangxi, prospective assessment of resource stability under future climate change remains a crucial task for ensuring energy security and optimizing strategic layouts. To this end, building on previous analyses, this study further introduces the LISA spatiotemporal transition method within the Exploratory Spatiotemporal Data Analysis (ESTDA) framework to quantify the potential migration risks of resources. By constructing a local Markov transition matrix, this method identifies the spatiotemporal transition types of local spatial association structures, thereby revealing the dynamic evolution paths and potential risks of solar energy resource distribution. The data used for ESTDA analysis in this study are the preprocessed annual total solar radiation amount data from 2000 to 2060. The calculation of global and local Moran’s I in the LISA spatiotemporal transition analysis was performed in GeoDa software, using the software’s default spatial weight matrix (Queen contiguity) and significance test parameters (p < 0.05). The subsequent identification of transition types was completed in Excel through quadrant rule judgement based on LISA clustering results (i.e., types such as high-high, low-low, high-low, low-high, and non-significant).

#### Model enhancements and uncertainty analysis

##### IGTWR model with a Bayesian framework

To test the statistical robustness and reliability of the IGTWR model estimation results in different periods under various scenarios, this study adopted an optimized Bayesian statistical method to systematically validate the model coefficients.

##### Sensitivity analysis of the enhanced IGTWR model

To test IGTWR robustness under data perturbations, we conducted a sensitivity analysis. Annual data of 550 nm aerosol optical depth from 2026 to 2060 under the SSP1-2.6 scenario were selected, and a global perturbation of ±10% was applied to their observed values. Based on the coefficient results before and after perturbation, three indicators—variation amplitude, average absolute change rate, and stability score—were used for quantitative evaluation.

##### ESTDA with a Markov chain and Monte Carlo framework

In addition to the potential issues of the IGTWR model, although the ESTDA framework based on LISA relative length can effectively characterize the spatiotemporal migration risks of solar energy resources, its descriptive conclusions are insufficient to meet the needs of prospective risk assessment. Therefore, to quantify abstract evolution patterns into probabilizable risk indicators and provide a statistical inference basis for uncertainty analysis, this study introduces a probabilistic risk assessment framework that integrates Markov chains and Bootstrap Monte Carlo simulation.

### Quantification and statistical analysis

Data Presentation in Figures: In figures presenting summary data, values are shown as the arithmetic mean. Error bars represent the standard error of the mean (SEM), as indicated in the respective figure legends. The sample size (n = 14) for each aggregated data point (i.e., the annual mean across 14 prefecture-level cities) is explicitly stated in the legends.

All statistical analyses and quantifications in this study were conducted based on the following methods and parameters. Unless otherwise specified, the statistical significance thresholds were set at p < 0.01, p < 0.05, and p < 0.1.

Trend analysis: The Mann-Kendall non-parametric trend test and the Theil-Sen trend estimator were adopted.Given that the Mann-Kendall test is robust to non-normal data distributions and outliers, no additional normality verification was performed for this analysis.

Spatial autocorrelation analysis: Global and local Moran’s I were calculated in GeoDa software. The Queen spatial weight matrix was used, and significance was evaluated through 999 permutation tests.Prior to analysis, the consistency of spatial data projection and resolution was verified to ensure compliance with the spatial autocorrelation test assumptions.

Driver factor screening: The Optimal Parameter-based Geographical Detector (OPGD) was used, with the q-statistic as the indicator to measure the explanatory power of factors. No special distributional assumptions are required for OPGD, so no additional hypothesis verification was conducted. The construction of the IGTWR model and goodness-of-fit evaluation (including VIF, R^2^, adjusted R^2^, and AICc) were completed in the aegis toolset of the ArcMap 10.8.2 platform. For the IGTWR model, multicollinearity was tested using the variance inflation factor (VIF), with all VIF values < 10, indicating no significant multicollinearity among driving factors and meeting the model’s assumption of no severe collinearity.

#### Model robustness and uncertainty

Robustness test: A Bayesian statistical method was adopted to assess the stability of model coefficients by estimating their posterior distributions. The convergence of the Bayesian Markov Chain Monte Carlo (MCMC) simulation was verified using the Gelman-Rubin diagnostic (potential scale reduction factor < 1.05), confirming that the posterior distribution estimates are reliable.

Sensitivity analysis: A global perturbation of ±10% was applied to key input variables (550 nm aerosol optical depth data), and quantitative evaluation was performed using the following three indicators: variation amplitude, average absolute change rate, and stability score.No additional hypothesis verification was required for this analysis, as it focuses on the response of model outputs to input perturbations.

Probabilistic risk assessment: Bootstrap Monte Carlo simulation was used with 300 resampling iterations to calculate the probability distributions and confidence intervals of risk indicators. The normality of the resampled risk indicator distributions was verified using the Shapiro-Wilk test, and appropriate statistical methods were selected based on the verification results for interval estimation.

Software and implementation: Analyses were conducted using MATLAB R2024a, RStudio, GeoDa, and ArcMap 10.8.2, respectively.All custom codes have been made publicly available.

## Supplemental information

Document S1. Tables S1–S7

Document S2. Data S1–S6 Data S1: Code for statistical downscaling based on k-nearest neighbor search with Delta bias correction. Data S2: Code for the Mann-Kendall and Theil-Sen (MK-TS) trend test. Data S3: Code for rotated empirical orthogonal function (REOF) analysis. Data S4: Code for the optimal parameter-based geographical detector (OPGD). Data S5: Code for the robustness test for the interactive geographically and temporally weighted regression (IGTWR) model. Data S6: Code for exploratory spatiotemporal data analysis (ESTDA) based on Markov chain and Monte Carlo methods.
