# Supplementary material for: Hybridization of CMIP6 and spatiotemporal models for assessing solar energy dynamics and transition risks in Guangxi under “dual-carbon” goals
Source: iScience. 2026 Jan 14;29(2):114690. doi: 10.1016/j.isci.2026.114690 (PMC12886514; doi:10.1016/j.isci.2026.114690)
Supplement: Document S1. Tables S1–S7 [file mmc1.pdf]

iScience, Volume *29*

Supplemental information

Hybridization of CMIP6 and spatiotemporal models for assessing solar energy dynamics and transition risks in Guangxi under "dual-carbon" goals

Yisong Han, Xiangling Tang, Wei Li, and Siyi Hu

Table S1. Comparison and validation results between ERA5 reanalysis data and station observational data (1980–2023), related to Figure 1

Validation Variable	Unit	Correlation Coefficient	MAE	RMSE	Number of Valid Stations	Data Quality
Near-Surface Air Radiation (rsds)	$J \cdot m^{-2} \cdot a^{-1}$	0.98	37.22	44.56	44	Correlation coefficient > 0.95, error proportion < 1%
Total Cloud Cover Percentage (clt)	%	0.86	1.02	1.19	44	Correlation coefficient > 0.85, error proportion < 5%
Surface Air Pressure (ps)	Pa	0.99	1.31	2.34	44	Correlation coefficient > 0.95, error proportion < 1%
Near-Surface Air Temperature (tas)	K	0.85	0.74	1.18	44	Correlation coefficient > 0.80, error proportion < 5%
Near-Surface Relative Humidity (hurs)	%	0.90	0.57	0.76	44	Correlation coefficient > 0.90, error proportion < 1%

Table S2. Comparison and validation results between bias-corrected and downscaled CMIP6 data and station observational data (1980–2023), related to Figure 1

Validation Variable	Unit	Correlation Coefficient	MAE	RMSE	Number of Valid Stations	Data Quality
Near-Surface Air Radiation (rsds)	$J \cdot m^{-2} \cdot a^{-1}$	0.96	38.65	45.12	44	Correlation coefficient > 0.95, error proportion < 1%
Total Cloud Cover Percentage (clt)	%	0.86	1.64	1.42	44	Correlation coefficient > 0.85, error proportion < 5%
Surface Air Pressure (ps)	Pa	0.97	1.56	2.75	44	Correlation coefficient > 0.95, error proportion < 1%
Near-Surface Air Temperature (tas)	K	0.83	0.82	1.67	44	Correlation coefficient > 0.80, error proportion < 5%
Near-Surface Relative Humidity (hurs)	%	0.88	0.62	0.92	44	Correlation coefficient > 0.85, error proportion < 1%

Table S3. Statistical Significance Test Results of the Dominant REOF Modes of Solar Radiation in Guangxi Under the SSP1-2.6 Scenario (Based on the North Criterion), related to Figure 5

Scenario & Period	Mode	Eigenvalue (λ)	Variance Explained (%)	Sampling Error (±δλ)	Error Range	Significantly Separated?
SSP1-2.6 (2025-2030)	1	15.1	42.01	1.39	(13.71, 16.49)	Yes
	2	9.9	27.41	1.25	(8.65, 11.15)	No
SSP1-2.6 (2031-2060)	1	17.2	47.78	1.48	(15.72, 18.68)	Yes
	2	7.8	21.67	1.14	(6.66, 8.94)	No

Table S4. Statistical Significance Test Results of the Dominant REOF Modes of Solar Radiation in Guangxi Under the SSP2-4.5 Scenario (Based on the North Criterion), related to Figure 6

Scenario & Period	Mode	Eigenvalue (λ)	Variance Explained (%)	Sampling Error (±δλ)	Error Range	Significantly Separated?
SSP2-4.5 (2025-2030)	1	15.9	44.17	1.41	(14.49, 17.31)	Yes
	2	9.2	25.56	1.2	(8.00, 10.40)	No
SSP2-4.5 (2031-2060)	1	16.8	46.67	1.46	(15.34, 18.26)	Yes
	2	8.5	23.61	1.18	(7.32, 9.68)	No

Table S5. Statistical Significance Test Results of the Dominant REOF Modes of Solar Radiation in Guangxi Under the SSP3-7.0 Scenario (Based on the North Criterion), related to Figure 7

Scenario & Period	Mode	Eigenvalue (λ)	Variance Explained (%)	Sampling Error (±δλ)	Error Range	Significantly Separated?
SSP3-7.0 (2025-2030)	1	14.5	40.28	1.36	(13.14, 15.86)	Yes
	2	10.1	28.06	1.26	(8.84, 11.36)	No
SSP3-7.0 (2031-2060)	1	13.2	36.67	1.3	(11.90, 14.50)	Yes
	2	9.6	26.67	1.24	(8.36, 10.84)	No

Table S6. Summary of Robustness Analysis Results of the IGTWR Model, related to STAR Methods

Scenario/ Period	Variable 1	Variable 2	Variable 3	Variable 4	Interaction Consistency	Number of Robust Variables	Overall Evaluation
SSP1-2.6 (2025-2030)	Very Strong (2310.85)	Very Strong (141.19)	Moderate (6.20)	Very Strong (447815.73)	Limited (P=0.006)	3/4	Excellent
SSP1-2.6 (2031-2060)	Strong (24.14)	Extreme (1.18e+27)	Strong (15.35)	Extreme (6.86e+140)	Limited (P=0.000)	2/4	Mixed (but with Extreme Evidence)
SSP2-4.5 (2025-2030)	Extreme (1.88e+133)	Very Strong (1111.85)	Moderate (9.03)	Strong (28.69)	Not Robust (P=0.395)	2/4	Moderate
SSP2-4.5 (2031-2060)	Very Strong (6357.84)	Strong (22.44)	Very Strong (24611.51)	Extreme (Inf)	Limited (P=0.000)	3/4	Excellent
SSP3-7.0 (2025-2030)	Insufficient Evidence (4.46)	Very Strong (3.34e+10)	Strong (59.56)	Strong (54.99)	Limited (P=0.090)	1/4	Poor
SSP3-7.0 (2031-2060)	Very Strong (163.67)	Extreme (3.04e+63)	Strong (44.91)	Extreme (2.30e+140)	Limited (P=0.000)	3/4	Excellent

Table S7. Results of the IGTWR Model's Regression Coefficients for Cities in Guangxi After Input Data Perturbation, related to STAR Methods

Region	Baseline Coefficient	Coefficient After +10% Perturbation	Change Magnitude (+, %)	Coefficient After -10% Perturbation	Change Magnitude (-, %)	Average Absolute Change Rate (%)	Stability Score
Nanning	-0.55	-0.58	+5.45	-0.53	-3.64	4.55	0.954
Liuzhou	-0.70	-0.74	+5.71	-0.67	-4.29	5.00	0.950
Guilin	-0.45	-0.47	+4.44	-0.43	-4.44	4.44	0.956
Beihai	-0.35	-0.36	+2.86	-0.34	-2.86	2.86	0.971
Yulin	-0.50	-0.53	+6.00	-0.48	-4.00	5.00	0.950
Baise	-0.40	-0.42	+5.00	-0.39	-2.50	3.75	0.962
Hechi	-0.38	-0.40	+5.26	-0.37	-2.63	3.95	0.961
Qinzhou	-0.33	-0.34	+3.03	-0.32	-3.03	3.03	0.970
Fangchenggang	-0.32	-0.33	+3.13	-0.31	-3.13	3.13	0.969
Guigang	-0.48	-0.51	+6.25	-0.46	-4.17	5.21	0.948
Wuzhou	-0.42	-0.44	+4.76	-0.41	-2.38	3.57	0.964
Laibin	-0.52	-0.55	+5.77	-0.50	-3.85	4.81	0.952
Hezhou	-0.37	-0.39	+5.41	-0.36	-2.70	4.05	0.959
Chongzuo	-0.36	-0.38	+5.56	-0.35	-2.78	4.17	0.958
